# Bubble biofilm: Bacterial colonization of air-air interface

**DOI:** 10.1016/j.bioflm.2020.100030

**Published:** 2020-06-16

**Authors:** Susanne Sjöberg, Courtney Stairs, Bert Allard, Rolf Hallberg, Felix Homa, Tom Martin, Thijs J.G. Ettema, Christophe Dupraz

**Affiliations:** a Department of Geological Sciences, Stockholm University, SE-106 91, Stockholm, Sweden; b Department of Cell and Molecular Biology, Uppsala University, Sweden; c Man-Technology-Environment Research Centre (MTM) Örebro University, SE-701 82, Örebro, Sweden; d Laboratory of Microbiology, Department of Agrotechnology and Food Sciences, Wageningen University, Stippeneng 4, 6708WE, Wageningen, the Netherlands

**Keywords:** Biofilm, Neuston, *Nevskia*, Air-air interface, Shallow subsurface, Ytterby mine

## Abstract

Microbial mats or biofilms are known to colonize a wide range of substrates in aquatic environments. These dense benthic communities efficiently recycle nutrients and often exhibit high tolerance to environmental stressors, characteristics that enable them to inhabit harsh ecological niches. In some special cases, floating biofilms form at the air-water interface residing on top of a hydrophobic microlayer. Here, we describe biofilms that reside at the air-air interface by forming gas bubbles (bubble biofilms) in the former Ytterby mine, Sweden. The bubbles are built by micrometer thick membrane-like biofilm that holds enough water to sustain microbial activity. Molecular identification shows that the biofilm communities are dominated by the neuston bacterium *Nevskia*. Gas bubbles contain mostly air with a slightly elevated concentration of carbon dioxide. Biofilm formation and development was monitored *in situ* using a time-lapse camera over one year, taking one image every second hour. The bubbles were stable over long periods of time (weeks, even months) and gas build-up occurred in pulses as if the bedrock suddenly exhaled. The result was however not a passive inflation of a dying biofilm becoming more fragile with time (as a result of overstretching of the organic material). To the contrary, microbial growth lead to a more robust, hydrophobic bubble biofilm that kept the bubbles inflated for extended periods (several weeks, and in some cases even months).

## Introduction

Benthic microbial communities typically organize themselves into biofilms or microbial mats, attached to a solid substrate. This ecological model of organization has been highly successful throughout Earth’s history with evidence of sedimentary microbial mats dating back to the Archaean time (e.g., Ref. [1]. Microbial mat communities are densely packed together in ecosystems where nutrients, electron donors, and acceptors are tightly and efficiently recycled (e.g., Ref. [2]. These communities are embedded in extracellular polymeric substances (EPS), acting as diffusion barriers that allow for a wide range of metabolic activities to coexist. The protective effect of EPS combined with highly flexible metabolisms strongly improves the tolerance of those communities to environmental stressors, explaining that we find microbial mats and biofilms in extreme environments.

In some instances, the hydrophobic nature of certain microbial EPS allows communities to colonize the air-water interface by forming floating biofilms [3,4]. Although such strategy is not fully understood, these aerobic communities seem to benefit from access to gaseous phases on one side and nutrients from the water on the other side [3]. Here, we document for the first time, bacterial communities that colonize the air-air interface by forming a peculiar ‘bubble biofilm’ attached to walls in tunnels leading to the main shaft of the former Ytterby mine [5,6]. Although it is unclear if these bubble biofilms represent a local curiosity or a larger ecological strategy, it provides another striking evidence of the extraordinary ability of the microbial world to adjust to any environmental challenge.

## Materials and methods

### The Ytterby mine area - site description

**Fig. 1:**
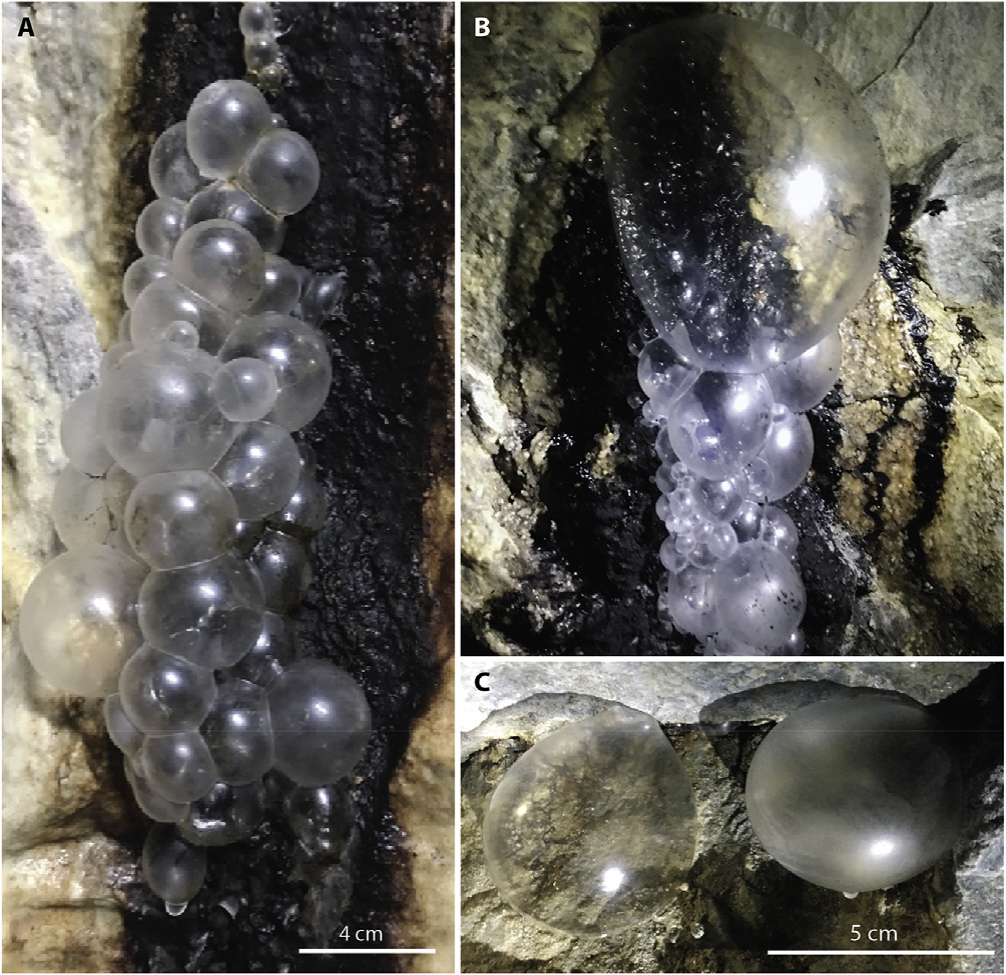
Bubble biofilms. (A and B) Gas trapped by bubble biofilm associated with a Mn oxide precipitates. (C) The difference in transparency reflects the level of maturity of the biofilm. The clear bubble is newly formed while the opaque one is more mature. There are small areas of initial Mn oxide precipitates on the rock wall inside both bubbles.

The former quartz and feldspar mine, also known for the discovery of tantalum and seven of the rare earth elements, is located on the shores of the Baltic Sea in the Stockholm archipelago, Sweden (59° 42’ 84″ N, 18° 35’ 38″ E). After closing in 1933, it reopened during the cold war era in the 1950s, to be used as a fuel deposit for the Swedish Armed Forces [5]. In connection with the reopening, a 400 ​m long tunnel system was built to link the old shaft to a newly constructed quay where ships were unloading the petroleum products. The studied gas-trapping bubble biofilm forms in association with water bearing rock fractures in these tunnels and covers surfaces of bedrock, carbonate travertine or manganese oxide precipitates (Fig. 1). The tunnels are situated 29 ​m below ground surface and 5 ​m above Baltic Sea mean sea level and holds a constant temperature of 8 ​°C all year round. Artifical lighting is used during mine maintenance, on average 2–3 ​h/month in the otherwise dark tunnel.

### Sample collection and DNA extraction

Samples for DNA extraction, amplification and sequencing were collected from fracture water and bubble biofilm covering Mn oxide precipitates associated with water bearing fractures in the mine tunnel. DNA was extracted from 0.5 ​g biofilm sample (n ​= ​4) using DNeasy PowerLyzer PowerSoil kit (Qiagen) and water filters (n ​= ​2) using DNeasy PowerLyzer PowerSoil kit (Qiagen). DNA extractions from the two water samples, each consisting of 160 ​mL water filtered through sterile 0.22 ​μm filter (Sterivex for sterile aqueous solutions), were conducted.

### *Small subunit rRNA gene amplification*, *sequencing and phylogenetic analysis*

Amplification of the targeted small subunit rRNA gene was conducted following a two-step PCR protocol using the universal primers’ combination: 519 forward and 1391 reverse as described in Spang et al. [7]; using HotStarTaq (Qiagen). Samples were sequenced on a MiSeq Illumina platform using Reagent kit v3, (600-cycle) at the SciLifeLab sequencing facility at Uppsala University, Sweden. Sequence features (here described as representative operational taxonomic unit, OTUs) were clustered at the 97% sequence identity level using QIIME2 (vsearch cluster-features-de-novo option). Taxonomic assignment of the OTUs was conducted using a naïve Bayesian classifier in QIIME2 (feature-classifier classify-sklearn) using a confidence score of 0.7 (--p-confidence) against the SILVA v132 database. To construct the phylogenetic tree, OTUs corresponding to the *Solimonadaceae* family were used as query sequences against the Genbank nucleotide database using BLASTN to retrive the top 20 sequences from each OTU. The resulting sequences were aligned using MAFFT-Q–INS–I [8] and the Ytterby OTUs were added using the “- - add” option. The alignment was trimmed to only include the amplicon region for both reference and Ytterby sequences. Maximum likelihood phylogenetic inference was conducted on the unmasked alignment with IQ-TREE v 2.0 [9] under the best scoring model of evolution selected with ModelFinder (TIM3+F ​+ ​I ​+ ​G4) with 1000 ultrafast bootstraps. Sequences obtained in this study were deposited in the NCBI Sequence Read Archive (SRA) as part of project number PRJNA544894. Sample accession numbers for the four biofilm samples are SAMN11898234, SAMN11898235, SAMN11898230 and SAMN11898229).

### Gas analyses

Gas trapped by bubble biofilm was sampled from four different locations: two samples of gas trapped by biofilm associated with a manganese oxide deposit (Fig. 1A, B), and two samples of gas trapped by biofilm of different maturity covering initial manganese oxide precipitates located in the same tunnel (Fig. 1C). Analyses of gases were conducted by Microbial Analytics Sweden AB. Three gas chromatograph systems equipped with three different detectors were used. Methane (CH_4_) ​> ​20 ​ppm, nitrogen (N_2_) and oxygen (O_2_) were partly analysed on a Varian CP-3800 gas chromatograph (Agilent Technologies Inc., CA, USA), equipped with a thermal conductivity detector (TCD) and high resolution capillary column (25 ​m∗0.53 ​mm ∗20 ​μm) CP7430 (Bruker, select permanent gases/CO_2_ HR) and partly on DANI Master GC, equipped with a TCD and column MXT-Molsieve 5A Plot (30 ​m∗0.53 ​mm∗50 ​μm). Carbon monoxide (CO) was also analysed using the latter system. Helium (He) was used as a carrier gas in both systems. CH_4_ and hydrocarbon gases (C1–C3) ​< ​20 ​ppm were partly analysed on Varian CP-3800 gas chromatograph equipped with a flame ionization detector (FID) and carboxen column (2 ​m∗1/8 in.∗2.1 ​mm) Ultimetal CP99969, with N_2_ as carrier gas and partly on Bruker 450 gas chromatograph (Bruker Daltonics, Scandinavia AB, Solna, Sweden) equipped with an PDHID detector (Valco Instruments Company, Inc, Houston, USA) and column PoraBOND Q (50 ​m∗0.53 ​mm, ID) CP7355, with He as carrier gas. Hydrogen (H_2_), oxygen and dinitrogen monoxide (N_2_O) were also analysed on a Bruker 450 gas chromatograph equipped with a PDHID detector and column MOLSIECW 5A PLOT (25 ​m∗ 0.32 ​mm, ID) CP7536. He was used as carrier gas. All chromatographs were calibrated using certified gas mixes (Air Liquide, Specialty gases, Krefeldt, Germay).

### Time lapse

*In situ* biofilm formation, development and gas trapping was monitored using a Brinno BCC200 professional time lapse camera. Time lapse imaging was conducted over one year, taking one image every second hour. Films are found in the supporting information (10.13039/100013443SI) and referred to in the text.

## Results

### Bubble biofilm formation

Bubble biofilm formation and development were monitored *in situ* using a Brinno BCC200 professional time-lapse camera over one year, taking one image every second hour. Time-lapse movies show that gas bubbles were stable over long periods of time (weeks, even months) and that gas build-up occurred in pulses as if the bedrock suddenly exhaled (SI 1). Over time, the bubbles matured and formed a robust hydrophobic biofilm that kept the bubbles inflated for extended periods (SI 2). No visible deflation was observed in the monitored bubbles, but occasional growth pulses. With time, the bubble biofilm migrated down the rock wall creating long chains or clusters of bubbles that occasionally shrank during the process due to mechanical strain (SI 3). Otherwise, only strong physical disturbances altered their evolution. For example, at times of high water supply, bubbles were washed down the bedrock and did not have time to settle (SI 4). Gas analyses show that bubbles contained mostly air with slightly elevated concentrations of carbon dioxide (Table 1). The average amount of carbon dioxide measured in the four bubbles, 517 ​ppm, was significantly higher (p˂0.025) than concentrations in the ambient tunnel air, 398 ​ppm.

**Table 1:** Analyses of gas trapped by biofilm (Bubbles 1 and 2, see Fig. 1A and B): gas trapped by biofilm on Mn deposit (Bubble 3, see Fig. 1C), immature biofilm bubble, and (Bubble 4, see Fig. 1C) mature biofilm bubble. (N_2_O, Ethane, Ethene, Ethyne, Propane, Propene, Propyne were also measured but only present in traces).

Gas (ppm)	Tunnel air	Bubble 1	Bubble 2	Bubble 3	Bubble 4
H_2_	<3.00	<3.00	<3.00	<3.00	<3.00
O_2_	173000	168000	176000	175000	174000
O_2_ ​+ ​Ar^a^	183000	177000	183000	189000	185000
N_2_	783000	788000	791000	781000	792000
CO	<20.00	<20.00	<20.00	<20.00	<20.00
CO_2_	398	563	435	574	496
CH_4_	13.6	10.8	12.0	13.0	19.9

a High oxygen levels make it difficult to separate oxygen from argon. Therefore oxygen is also reported as argon ​+ ​oxygen as a combined peak. The remaining gas, to receive 100% analysed gas, could be helium that was not analysed.

### Microbial community composition

**Fig. 2:**
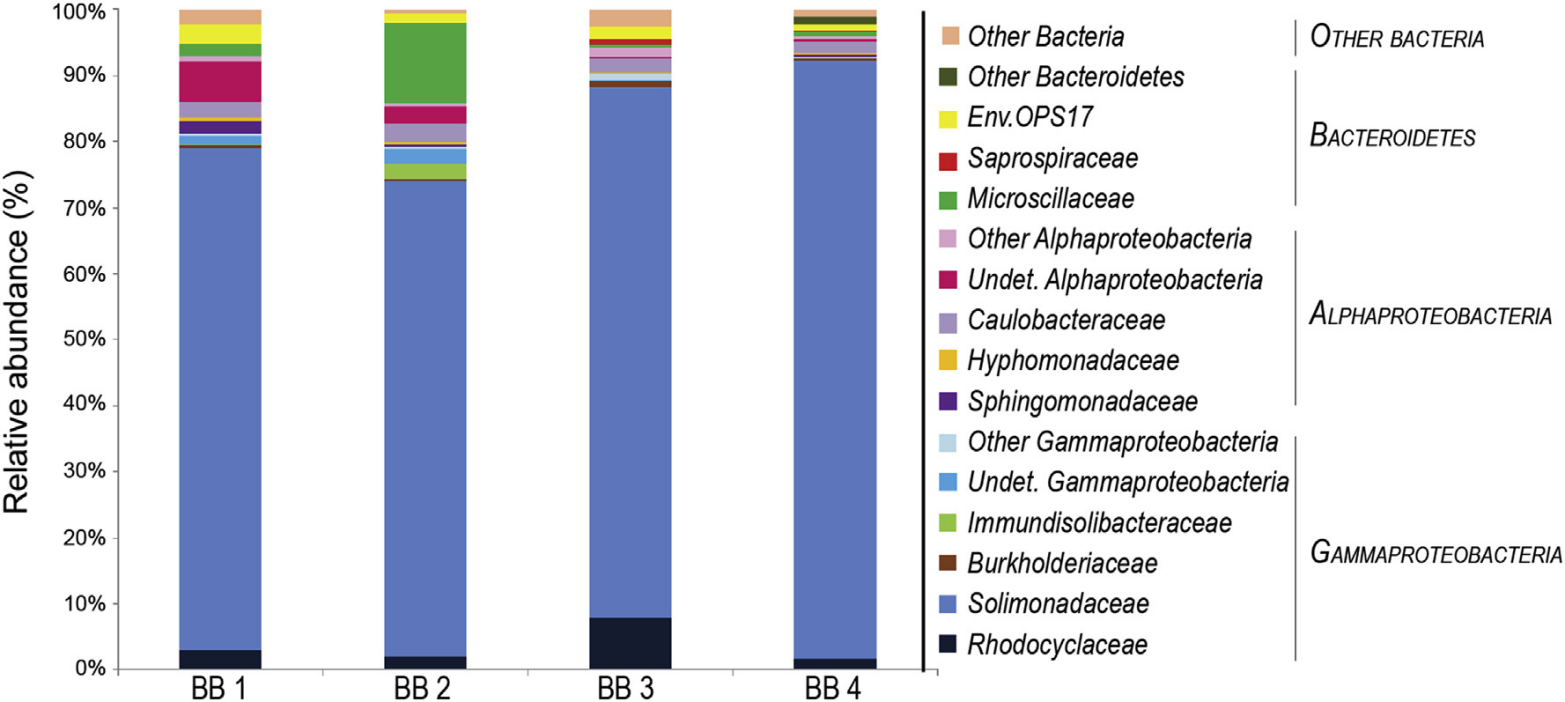
Bacterial community composition based on 16S rRNA gene analyses of the four bubble biofilm samples. BB 1, BB 2, BB 3 and BB 4 correspond to NCBI sample accession numbers SAMN11898234, SAMN11898235, SAMN11898230 and SAMN11898229, respectively. Percentages show the relative abundance of the bacterial community based on the frequency of bacterial 16S rRNA in each samle. Taxonomic assignment is made at family level.

**Fig. 3:**
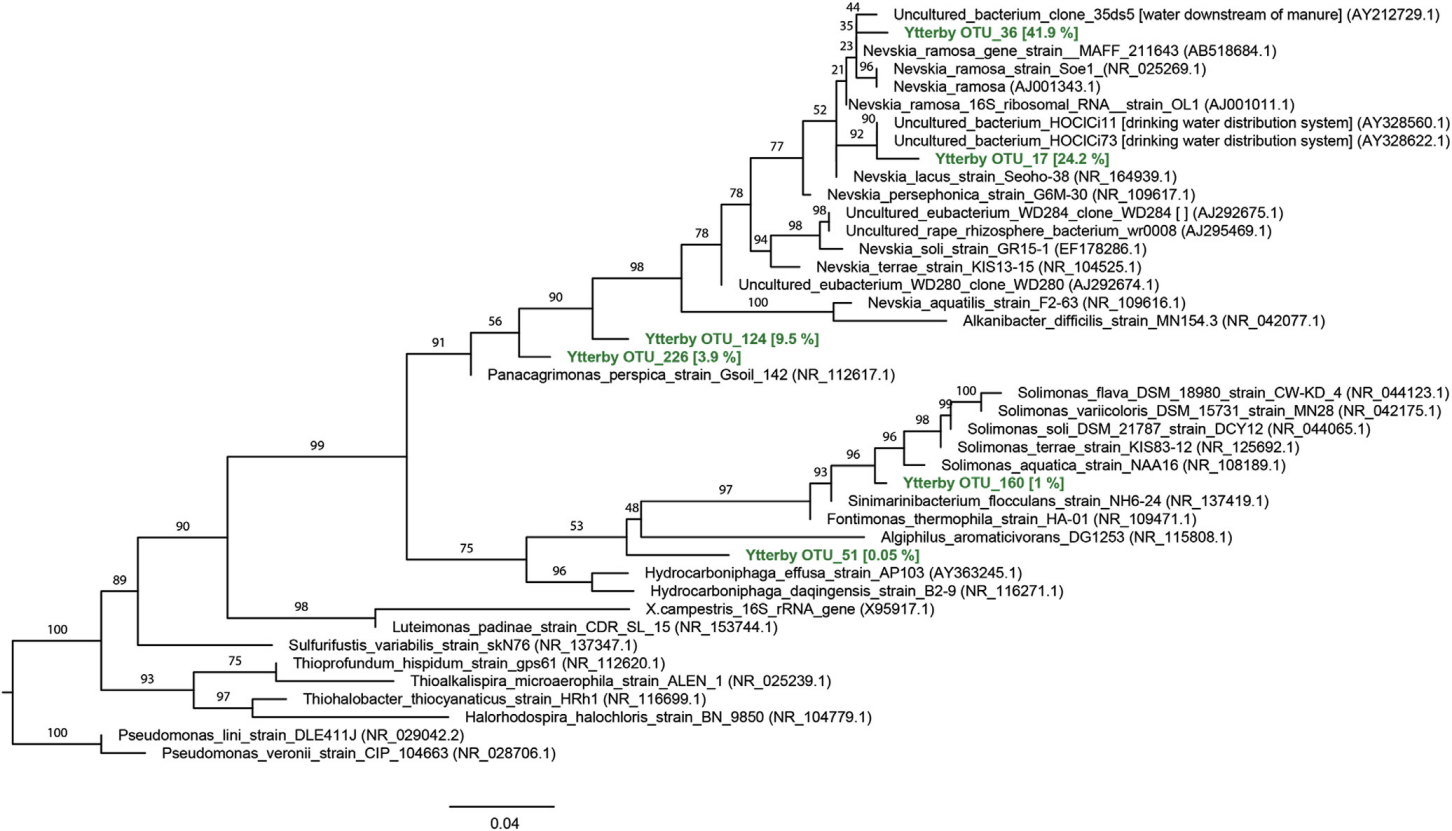
Maximum-likelihood phylogeny of the 16S rRNA gene sequences of the Solimonadaceae family from reference (black) and this study (green). Bipartition supports corresponding to ultrafast bootstraps are shown. The average relative abundance of the amplicons in across the four samples presented here is given in square brackets (sequence reads are given in SI. 5). Only bootstrap values above 50% are given at branch nodes. The scale bar represents the number of substitutions per unit branch length. (For interpretation of the references to colour in this figure legend, the reader is referred to the Web version of this article.)

DNA analyses indicated that all bubble biofilm samples were dominated by members of the *Gammaproteobacteria* class (between 79% and 93% of the total prokaryotic community), in which sequences belonging to the *Solimonadaceae* family and in particular the *Nevskia* genus were predominant (Fig. 2). The relative abundance of *Nevskia* in the fracture water (feeding the system) was very low: 0.9% 16S rRNA gene reads compared to an average of 65.9% in the biofilm samples. This group of *Nevskia* bacteria clustered into two OTUs: one OTU is 97.76% similar to *Nevskia ramosa* strain Soe1 DSM 11499 (NR_025269 [10], and the second OTU is 98.9% similar to *Nevskia ramosa* strain MAFF 211643 (AB518684, Kawai, NCBI GenBank 2019). Within the same family there was also a high relative abundance of sequences belonging to the *Panacagrimonas* genus. A phylogenetic tree was constructed to show the position of *Solimonadaceae* sequences obtained in this work (Fig. 3).

In general there was little variation among the four biofilm samples with the exception of the *Bacteroidetes* and *Alphaproteobacteria*. In the *Bacteroidetes* group there were substantial differences in terms of relative abundance (ranging from 0.2% to 12% of the 16S rRNA gene reads). Sequences mainly clustered within the *Microscillaceae* family which is a group of chemoorganotrophic, strictly aerobic bacteria that are capable of gliding motility [11]. Within the Alphaproteobacteria there were also differences in relative abundance between the samples (ranging from 3.2% to 11.7% of the 16S rRNA gene reads), but here the groups that contributed most to these differences remained undetermined.

## Discussion

When growing undisturbed, *Nevskia* form microcolonies at the air-freshwater interface that display a rosette- or bush-like morphology [12,13]. These strictly chemoorganotrophic aerobes are mainly found in shallow aquatic environments such as swamps, ponds, pools, and so forth (see references in Ref. [12]. They are defined as epineuston, *i.e.*, organisms that float on the water surface. Communities are mainly located outside the water phase where they reside on top of a hydrophobic microlayer that develops on the water surface [10,12,14]. The result is an opaque pellicle (floating biofilm), with a hydrophobic nature similar to that of paraffin [15].

**Fig. 4:**
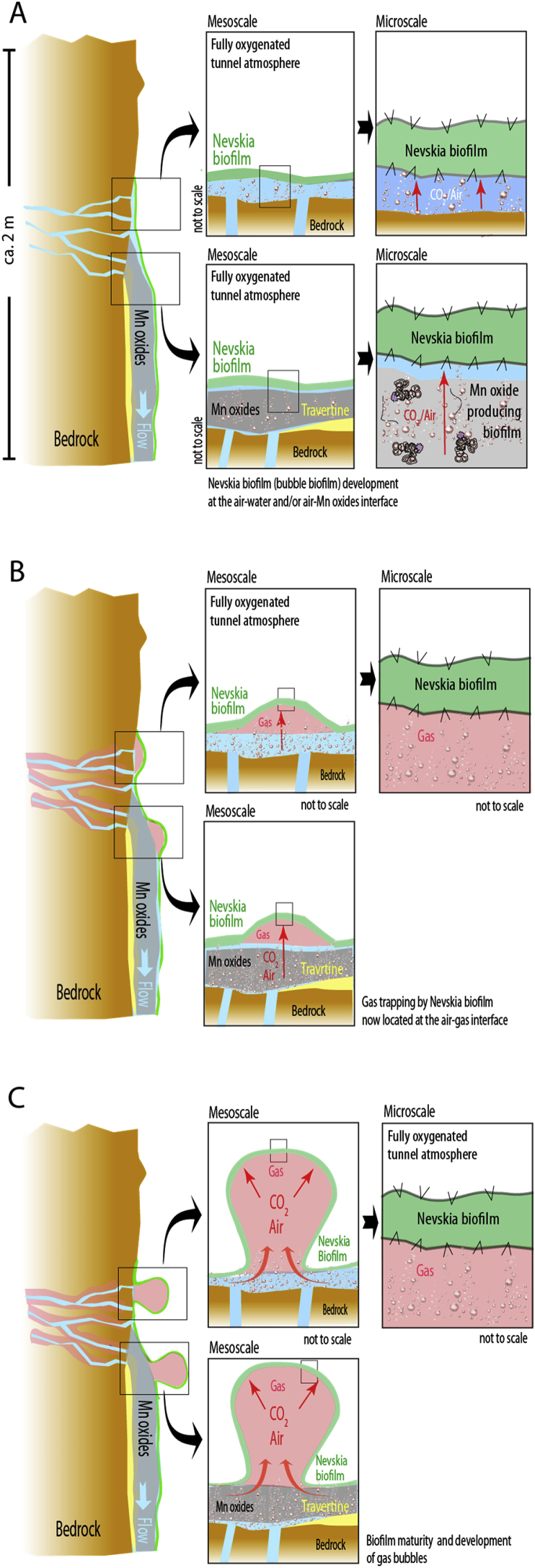
Model of bubble biofilm formation (A) Due to its partial hydrophobic nature, the *Nevskia* dominated biofilm colonize the air-water interface attached to bedrock surfaces, lithified calcium carbonate (travertine), or Mn precipitates. (B) Groundwater reaching the bedrock surface releases pockets of air that have been trapped inside the water bearing rock fractures and also equilibrates with the tunnel atmosphere which induces CO_2_ degassing. This gas diffuses through the Mn oxides but not through (or very slow diffusion) the *Nevskia* biofilm, contributing to formation of gas bubbles. (C) Gas build-up (increase in bubble size) occurs in periodic pulses. The bubble then remains stable for several months (no visible deflation but occasional growth pulses). Over time, the bubble migrate down the rock wall (the first bubble moves downward when a new bubble is formed) creating long chains or cluster of bubbles. Mechanical disruption of the biofilm during migration (from rolling down on an irregular substrate) occasionally leads to gas losses. Although *Nevskia* bacteria are situated at the air-air interface, the biofilm still holds sufficient water to sustain microbial activity and biofilm maturation.

In contrast, the *Nevskia* dominating biofilm in Ytterby does not reside entirely at the boundary between water and air but rather at the bubble gas-air interface Fig. 4. It creates a micrometer size membrane-like biofilm in contact with air on both sides, but still holding sufficient water to sustain microbial activity. Indeed, the result is not a passive inflation of a dying biofilm becoming more fragile with time (as a result of overstretching of the organic material). It instead represents a continuous microbial growth that produces a thicker, more robust, and less transparent bubble biofilm, indicating a more mature stage (Fig. 1C). Although the trapped gas mainly reflects the ambient tunnel air, pressure has to be slightly higher inside the bubble to keep it inflated.

The stable nature of these bubbles can either be explained by (1) a constant influx of gas compensating the possible diffusion losses through the biofilm, or (2) a biofilm that is gas-tight and does not allow for much diffusion over time. In option one, the gas would either be produced by microbial activity or by groundwater CO_2_ degassing (reequilibration of the recharge fracture water with the tunnel air following Henry’s law). In those cases, we would expect an enrichment in a specific gas within the bubble atmosphere (e.g., CO_2_, CH_4_, H_2_). In our measurements we only record a minor elevation in CO_2_. The enrichment could theoretically also be prevented by the equilibration with the tunnel air through a gas permeable biofilm. However, the observation that gas builds up in pulses, rules out this hypothesis which would imply a constant and/or gradual inflow. We therefore favor the second option which assumes a biofilm that is creating a tight barrier that not even gas can escape (or very slow diffusion). The source of the gas could be a release of air pockets that have been trapped inside the water bearing rock fractures.

In a scenario involving a gas-tight biofilm trapping pockets of air originating from the bedrock fractures, the gas itself does not appear to be an essential factor in this system. It merely spatially separates the *Nevskia* biofilm that initially was situated at the air-water interface from the other underlying substrate (e.g., bedrock, travertine carbonates, and manganese oxide precipitates). Other communities associated with these substrates let the gas diffuse through while the *Nevskia* biofilm traps it. The possible selective advantages by colonizing the air-air interface are, however, not yet well understood and the function of the bubble biofilm in this underground ecosystem needs to be further investigated. Recently lipid profile changes have proven to be a useful tool for investigating microbial adaptations under harsh conditions [16]. We therefore intend to conduct analyses of lipids in the bubble biofilm at different maturity stages and also compare these profiles to those of *Nevskia* biofilms found in the literature and/or in other environments. Whether the bubble biofilm represents an opportunistic local curiosity or a new ecological colonization strategy, it provides another striking example of the fantastic ability of the microbial world to adjust to any environmental challenge.

## CRediT authorship contribution statement

**Susanne Sjöberg:** Conceptualization, Data curation, Formal analysis, Funding acquisition, Investigation, Methodology, Project administration, Writing - original draft, Writing - review & editing. **Courtney Stairs:** Data curation, Funding acquisition, Writing - review & editing. **Bert Allard:** Writing - review & editing. **Rolf Hallberg:** Methodology, Writing - review & editing. **Felix Homa:** Data curation. **Tom Martin:** Supervision, Writing - review & editing. **Thijs J.G. Ettema:** Funding acquisition, Supervision, Writing - review & editing. **Christophe Dupraz:** Conceptualization, Formal analysis, Funding acquisition, Investigation, Methodology, Writing - original draft, Writing - review & editing.

## Declaration of competing interest

The authors declare that they have no conflict of interest. This article does not contain any studies involving animals or human participants performed by any of the authors.
