# Supplementary material for: Bubble biofilm: Bacterial colonization of air-air interface
Source: Biofilm. 2020 Jun 16;2:100030. doi: 10.1016/j.bioflm.2020.100030 (PMC7798470; doi:10.1016/j.bioflm.2020.100030)
Supplement: Multimedia component 5 [file mmc5.docx]

**SI. 5**
